# Sonographic and Clinicopathological Characterization of Struma Ovarii: A Retrospective Analysis for Enhanced Preoperative Diagnosis

**DOI:** 10.2174/0115734056393689250929185251

**Published:** 2025-10-10

**Authors:** Jianlin Cao, Zhaowei Meng, Cuimei Li

**Affiliations:** 1 Department of Nuclear Medicine, Tianjin Key Lab of Functional Imaging & Tianjin Institute of Radiology, Tianjin Medical University General Hospital, Tianjin 300052, China; 2 Department of Gynecology and Obstetrics, Shanxi Provincial People’s Hospital, Shanxi, China; 3 Department of Gynecology and Obstetrics, Xi'An Fifth Hospital, Xi'An, 710000 Shaanxi, China

**Keywords:** Ovary, Struma ovarii, Ultrasound, Pathology, Prognosis, CT, MRI, Carcinoembryonic antigen

## Abstract

**Introduction:**

Struma ovarii (SO) is a rare ovarian teratoma composed predominantly of thyroid tissue, often misdiagnosed due to its non-specific clinical manifestations and low prevalence.

**Methods:**

The ultrasound and clinical features of 16 histologically confirmed cases of SO (mean age 45 ± 10 years) were analyzed. Key ultrasound parameters evaluated included tumor size, internal echo patterns, calcification, blood flow (Adler grading), and pelvic effusion.

**Results:**

Half of patients with SO have been found to be postmenopausal women over 50 years of age, and that most tumors are discovered incidentally during routine examination. The large cystic components with regular margins, accompanied by calcified and vascularized solid elements, are ultrasound characteristics of SO. In particular, the presence of calcification and distinct vascular patterns on Doppler imaging (as per Adler classification) has been identified as a critical marker distinguishing SO from other adnexal masses.

**Discussion:**

Compared to existing SO research, this study has found the ultrasound characteristics of SO to mostly manifest as a large cystic echo, regular boundaries, and calcification. At the same time, compared to the existing imaging techniques, such as CT and MRI, characteristic ultrasonography has been found to be a good complement to the diagnosis of SO.

**Conclusion:**

When an adnexal tumor is classified as O-RADS 3-5 and exhibits features, such as a large cystic echo, regular boundaries, and calcification, SO should be considered in the differential diagnosis. These findings can enhance the accuracy of preoperative assessment, facilitate individualized surgical planning, and contribute to improved clinical management by reducing the likelihood of misdiagnosis.

## 1 INTRODUCTION

Struma ovarii (SO) is a rare ovarian tumor characterized by the presence of thyroid tissue comprising at least 50% of the ovarian mass. It is considered a specialized form of ovarian teratoma and may occasionally be associated with other epithelialcomponents,suchasmucinousorserous cystadenoma [1, 2]. Teratomas containing predominantly thyroid tissue or harboring malignant thyroid elements are classified as SO [3]. SO accounts for approximately 1% of all ovarian tumors and 2-5% of ovarian teratomas.

Due to its rarity and non-specific clinical presentation, SO is frequently misdiagnosed. Most patients are asymptomatic, and the tumor is often discovered incidentally during routine physical examinations or imaging for unrelated conditions. When diagnosed, the standard treatment involves surgical resection, typically followed by adjuvant radioiodine therapy to minimize the risk of recurrence or metastatic spread [4].

Ultrasound is widely used in clinical diagnostics due to its non-invasive nature and absence of radiation exposure. However, current research on SO remains limited, focusing primarily on case reports and small clinical studies, with relatively little attention given to its sonographic features [5-7]. This study aimed to address this gap by investigating the ultrasound characteristics of SO, analyzing its clinico-pathological features, and enhancing the accuracy of preoperative evaluation.

## 2 MATERIAL AND METHODS

### 2.1 Purpose

This retrospective study analyzed 16 patients aged 25 to 60 years (mean 45 ± 10) with unilateral, pathologically confirmed SO lesions who underwent preoperative ultrasound examination at Shanxi Provincial People's Hospital between January 2017 and March 2023. The study was approved by the Ethics Committee of Shanxi Provincial People's Hospital (approval no.: 2022/321), with a waiver for written informed consent.

### 2.2 Instruments and Methods

Ultrasound examinations were performed using GE Voluson E8 and E10 diagnostic instruments equipped with RIC5-9-D and CI-5-D probes. For transvaginal ultrasound, the probe frequency ranged from 5.0 to 9.0 MHz, and patients were placed in the lithotomy position with an emptied bladder. For transabdominal ultrasound, the probe frequency ranged from 3.5 to 5.0 MHz, and patients were positioned supine with a suitably filled bladder.

### 2.3 Methods

A comprehensive ultrasound examination was performed for each patient, during which the uterus and adnexa were carefully scanned in multiple planes to ensure thorough assessment. Once a tumor was identified in the adnexal region, a detailed evaluation was conducted to document its key characteristics, including length, width, precise location, internal echogenicity, boundary definition, overall shape, vascularity, and its anatomical relationship with surrounding tissues. In addition to transvaginal scanning, an abdominal probe was utilized to examine the entire pelvis and abdomen, allowing for the detection of additional pelvic or abdominal masses as well as the presence of ascites. Alongside the imaging findings, extensive clinical data were collected for each patient. These included demographic information, such as age and reproductive history, laboratory results, such as tumor markers and thyroid function tests, as well as definitive pathological diagnoses and details of the surgical procedures performed.

The length of the lesion is defined as the largest diameter measured in the tumor's maximum cross-section, while the width refers to the widest diameter perpendicular to the length. The location of the tumor is categorized anatomically as either on the left or right side of the adnexa. The internal echogenicity of the tumor is classified into five distinct types based on the proportion of solid and cystic components: (1) completely solid, (2) solid-dominated (solid components comprising more than 75% of the mass), (3) mixed cystic-solid (solid components accounting for 25–75% of the mass), (4) cystic-dominated (cystic components comprising more than 75% of the mass), and (5) completely cystic.

**Fig. (1):**
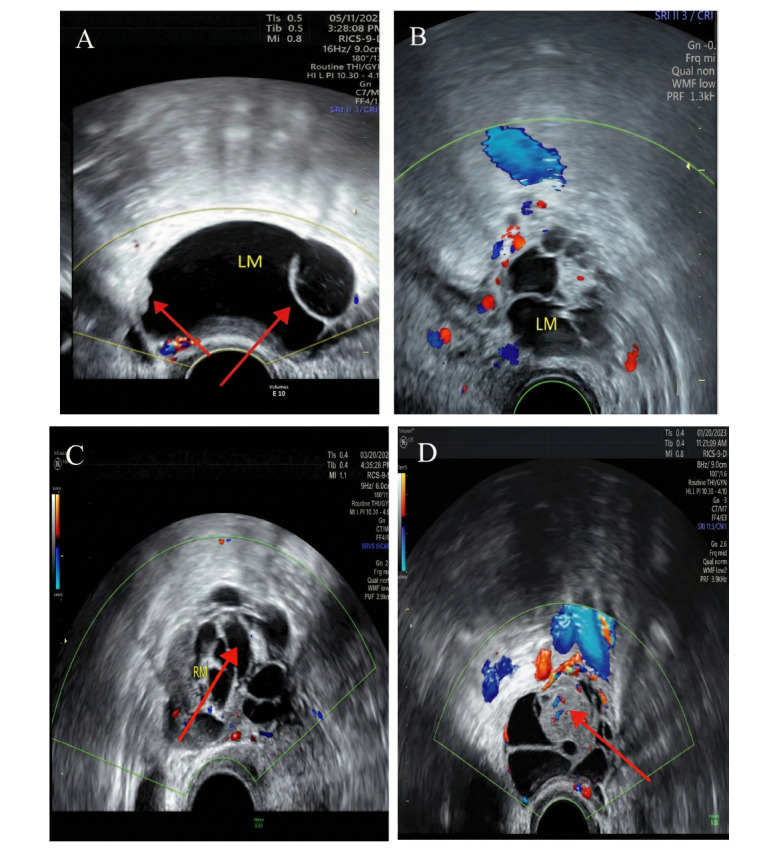
Ultrasonography of blood flow grading and internal septa (arrows) of ovarian mass. (**A**) Struma ovarii with blood flow grade 0. (**B**) Cystic mass with the blood flow grade I in the ovary. (**C**) Ovarian cystic mass with multi-septum and blood flow grade II. (**D**) Struma ovarii with cystic structures of various sizes in the left ovary, containing glial elements and raising the suspicion of goiter of the ovary. The interior is primarily cystic, and the blood flow grade is grade III.

The boundary of the lesion is assessed as either clear or unclear, and the shape is categorized as regular (round or oval) or irregular. To evaluate the tumor’s vascularity, the Adler grading method is employed: grade 0 indicates no detectable blood flow signals (Fig. **1A**); grade I shows a small amount of blood flow, characterized by 1-2 star-shaped or thin rod-like signals (Fig. **1B**); grade II represents moderate blood flow with 3-4 star-shaped signals or one long vessel (Fig. **1C**); and grade III reflects abundant blood flow, marked by more than four star-shaped signals, two or more long vessels, or an intricate network-like pattern observed within the mass (Fig. **1D**). In terms of effusion assessment, a rectouterine pouch (rectal fossa) fluid collection exceeding 5 mm is defined as pelvic effusion, whereas fluid detected in the iliac fossa is categorized as peritoneal effusion.

According to the REMARK checklist, the tumor markers assessed included carbohydrate antigen 125 (CA-125), carbohydrate antigen 19-9 (CA 19-9), carcinoembryonic antigen (CEA), and alpha-fetoprotein (AFP). The pathological classification of ovarian tumors was based on the 2014 Surgical Pathological Staging Guidelines established by the International Federation of Gynecology and Obstetrics (FIGO).

## 3 RESULTS

### 3.1 Basic Clinical Data, Ultrasonographic Characteristics, and Abnormal Blood Biochemical Indicators of SO Patients

**Fig. (2):**
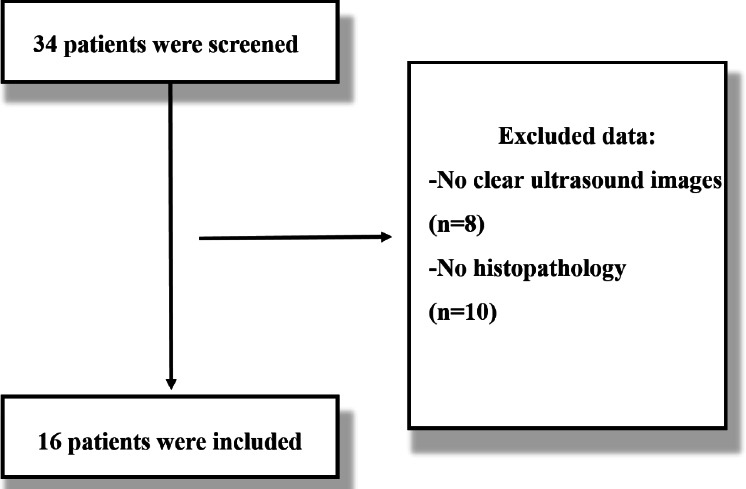
Flow diagram representing the selection of the patients.

**Fig. (3):**
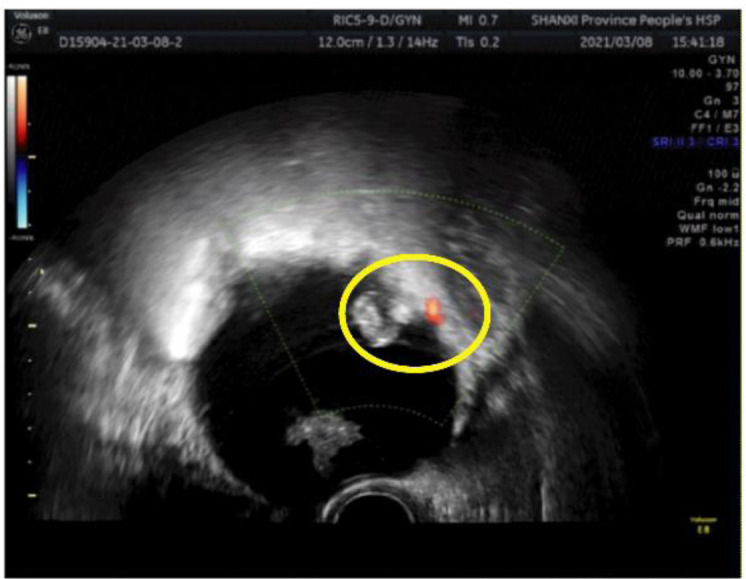
45-year-old patient with cystic lesion of the right ovary with 360° torsion. The cyst wall is lined with cuboidal epithelium. Less thyroid follicles can be seen in the focal cyst wall, and the boundary is clear. A hyperechoic mass can be seen inside, and the capsule wall can be seen by color Doppler. The yellow circles represent the dotted blood flow signal on the cyst wall.

In this study, 34 patients were initially screened, and after excluding those with unclear ultrasound images or incomplete pathological data, 16 female patients, ranging from 25 to 60 years old, with confirmed SO, comprising 16 lesions, were included for analysis (Fig. **2**). Of these, 9 cases were incidentally discovered during routine physical examinations, while the remaining 7 patients presented with symptoms of lower abdominal discomfort or pain. Tumor sizes varied widely, with maximum diameters ranging from 36 mm to 300 mm, and the majority were unilateral with a regular, round, or oval shape. Sonographic assessment showed that the internal echogenicity for the 16 tumors was predominantly cystic with solid components, with 14 of the 16 tumors displaying calcifications within the solid portions (Fig. **3**). Color Doppler imaging revealed internal blood flow in 12 of the 16 lesions, with 6 cases showing grade III flow, 5 cases grade I, and 1 case grade II based on the Adler grading method. Pelvic effusion was detected in 9 patients; among them, 1 also had peritoneal effusion, and another had both peritoneal and pleural effusions (Tables **1** and **2**). As CA125 is a commonly used biomarker for ovarian tumors, levels were measured in all patients, and elevated CA125 was observed in only 3 cases with cystic-solid lesions. To assess malignancy risk, all cases were evaluated using the Ovarian-Adnexal Reporting and Data System (O-RADS), resulting in 6, 8, and 2 patients being categorized as O-RADS 3, 4, and 5, respectively. Thyroid function testing revealed that one patient was positive for thyroid peroxidase antibody (42.2 IU/mL) and thyroglobulin antibody (213 U/mL), while another showed an elevated CA19-9 level (68.79 ng/mL).

**Fig. (4):**
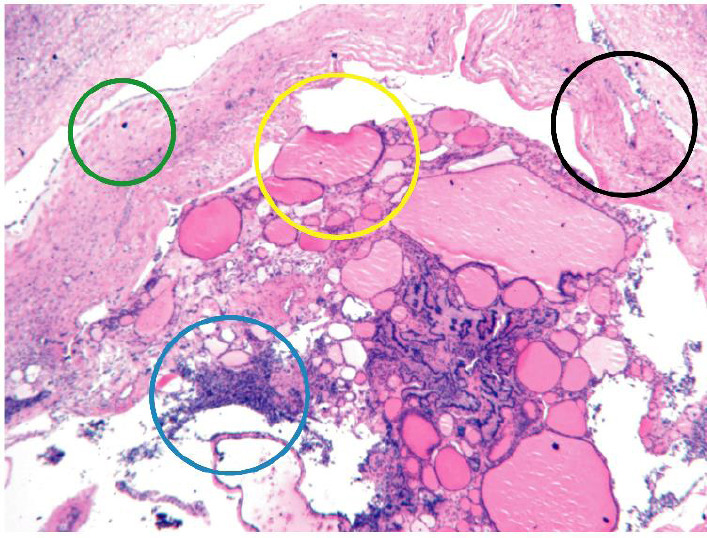
Follicles in struma ovarii. Yellow circles: thyroid follicular hyperplasia, varying sizes; blue circle: inflammatory cell infiltration; black circles: fibrosis and cystic changes; green circles: scattered calcifications. H&E*200.

**Fig. (5):**
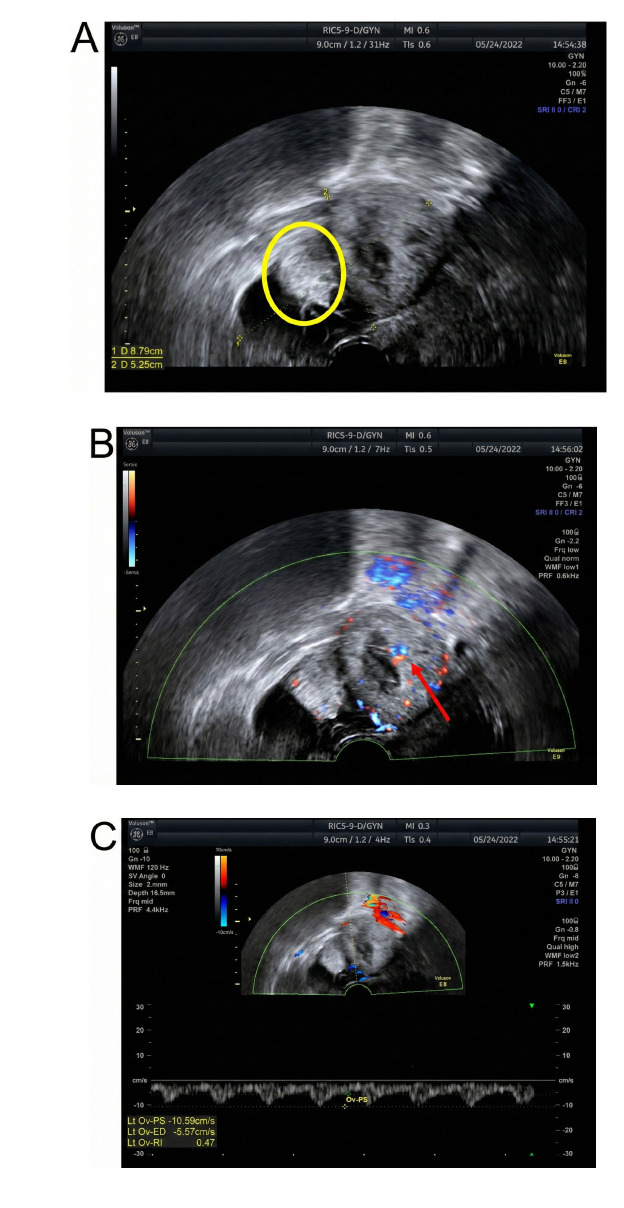
A 55-year-old female patient with struma ovarii. (**A**) The lesion exhibits clear boundaries, regular morphology, mainly solid mass, and visible calcification (yellow circles). (**B**) CDFI: The solid part of the lesion shows abundant blood flow signals (red arrow). (**C**) Color Doppler shows rich blood flow; RI: 0.47.

Pathological analysis of all 16 cases of SO revealed the presence of characteristic thyroid follicles in each section, with tumor diameters ranging from 4 to 28 cm (Fig. **4**). Immunohistochemical staining was performed in two cases, yielding the following results: one case demonstrated positive expression of TTF-1 (+), thyroglobulin (TG) (+), Ki-67 (+2%), MCK (+), CD56 (+), and thyroid peroxidase (TPO) (+); the other case showed positivity for CD56 (+), TPO (+), TG (+), and PAX-8 (+). Additionally, one patient was pathologically diagnosed with struma ovarii coexisting with follicular carcinoma of the thyroid. Immunohistochemistry in this case revealed approximately 10% positivity for Ki-67, diffuse staining for TTF-1 and TG, and positivity for TPO. Other notable histopathological features included hemorrhage in one case, presence of a scolex in one cystic lesion, and focal calcification in four cases (Fig. **5A**).

### 3.2 Operation Mode and Prognosis

The sixteen patients diagnosed with SO thereafter underwent laparoscopic surgery, with the specific procedures tailored to each case. One patient with ovarian thyroid carcinoma received a hysterectomy along with bilateral oophorectomy and omentectomy. Four postmenopausal patients underwent total hysterectomy with bilateral oophorectomy. Six patients had unilateral adnexectomy, while five underwent laparoscopic mass excision.

All patients were followed postoperatively for a duration ranging from six months to five years. During the follow-up period, no tumor recurrence was observed, and all patients remained alive and free from tumor-related complications.

## 4 DISCUSSION

This study analyzed the sonographic features and clinicopathological data of 16 histologically confirmed SO cases. While previous studies have reported SO as predominantly affecting women of reproductive age, our cohort demonstrated a distinct demographic trend, with half of the patients being postmenopausal women over the age of 50 and most tumors being detected incidentally during routine examinations [4]. Interestingly, cystic SO tended to occur in younger patients, with two cases (12.5%) identified in women under 30 years of age who presented either with incidental findings or symptoms of lower abdominal discomfort.

Ultrasound findings in this study revealed that the internal echoes of SO were predominantly well-defined cystic-solid in nature, with only three cases presenting as purely cystic lesions. Cystic tumors tended to be larger, with maximum diameters reaching up to 30 cm compared to 10 cm in cystic-solid lesions, and showed no detectable internal blood flow. Previous studies have described the sonographic features of SO as multilocular cystic or cystic-solid masses with “papillary”, “septal thickening”, or “dough-like” solid components, typically accompanied by abundant low-impedance blood flow signals (Fig. **5B** and **C**). While no blood flow is usually observed within the cystic cavities, they may contain “white ball-like” or “flocculent” hyperechoic masses, “crystalline” echogenic foci, and solid components, septations, or cyst walls with punctate, eggshell-like, or arc-shaped calcifications, which are suggestive of thyroid tissue involvement. Notably, the ultrasound appearance of SO may closely resemble that of ovarian serous or mucinous cystadenomas [8]. In cases where the tumor is not entirely composed of thyroid tissue or differentiation from mature teratoma is challenging, an iodine scan may aid in diagnosis. In our cohort, all 16 SO cases exhibited regular margins, and 14 demonstrated varying degrees of calcification, while the two purely cystic cases showed no significant calcification on two-dimensional ultrasound imaging [9, 10].

Additionally, the clinical symptoms of SO are closely related to tumor volume, with tumors of increasing size often accompanied by a palpable lower abdominal mass and abdominal pain due to compression of adjacent organs. In this study, the tumor volumes were relatively large, with the maximum diameter reaching 30 cm. On both ultrasound and intraoperative evaluation, the tumors typically appeared as regular, well-circumscribed masses [11]. The growth pattern of SO is primarily characterized by expansive rather than invasive behavior, which may be attributed to its non-epithelial cellular origin. A notable clinical manifestation associated with SO is pseudo-Meigs syndrome, a rare complication involving ascites and pleural effusion. In this study, two typical cases were identified. Ultrasound revealed serous effusions in the thoracic, abdominal, and pelvic cavities. Follow-up after surgical resection showed complete resolution of effusions within one month, confirming the reversible nature of SO-related Meigs syndrome. This finding underscores the importance of surgical management not only in removing the primary lesion, but also in alleviating associated serosal fluid accumulation. It is important to note that, due to the nonspecific imaging features and the presence of symptoms, such as ascites and elevated CA125, SO with Meigs syndrome may be misdiagnosed as an ovarian malignancy. In this cohort, one patient with pseudo-Meigs syndrome and an O-RADS score of 5 had their lesion overestimated in terms of malignancy. Although CA125 is a key biomarker in ovarian cancer diagnosis, its value in distinguishing benign from malignant SO is limited. In this study, CA125 levels were typically normal in SO cases, and although elevated in three patients, postoperative pathology confirmed benign disease. Therefore, definitive diagnosis relies on postoperative histopathological evaluation, which remains the gold standard.

In addition, this study identified 15 euthyroid patients with non-functional SO. Notably, one patient with coexisting thyroid follicular carcinoma was found to have autoimmune thyroiditis, evidenced by positive TPOAb and TgAb levels. Follow-up thyroid ultrasound revealed nodules in both lobes and the isthmus. Subsequent total thyroidectomy with central lymph node dissection uncovered an unexpected bifocal papillary thyroid carcinoma on pathological examination. This critical case has highlighted the importance of comprehensive thyroid evaluation in patients diagnosed with SO [12, 13].

Imaging evaluation of SO requires a comprehensive approach utilizing multiple modalities; therefore, for SO containing abundant thyroid tissue with high iodine uptake, the solid components may show marked enhancement on contrast-enhanced CT, closely resembling metastatic ovarian tumors and thereby complicating the diagnostic process. In contrast, while MRI offers superior soft tissue resolution [14, 15], ultrasound provides real-time imaging, is more cost-effective, involves no radiation exposure, and enables assessment of vascularity, making it particularly advantageous in clinical settings [16]. Despite the strengths of each modality, no imaging technique can reliably distinguish benign from malignant SO preoperatively; thus, definitive diagnosis continues to rely on histopathological examination [17, 18].

This study’s proposed methodology offered several key advantages. A comprehensive evaluation identified that the presence of large cystic components alongside calcified and vascularized solid elements served as an independent diagnostic feature of SO, effectively distinguishing it from other ovarian neoplasms. Importantly, the findings reinforced the value of ultrasound as a critical diagnostic modality, with calcification and characteristic vascular patterns, particularly as classified by the Adler system, emerging as significant discriminative markers. These insights contributed to improved preoperative diagnostic accuracy, guided surgical decision-making, and ultimately enhanced clinical management of patients with SO. However, this study has involved several limitations. The small sample size from a single center limited the generalizability of the findings. The retrospective study design and reliance on previously published literature may have introduced bias, particularly in estimating recurrence rates. Furthermore, the absence of genetic or molecular analysis restricted insight into potential biological factors associated with recurrence and malignant transformation [17, 19-24]. Future multicenter studies with larger patient cohorts are needed to validate these findings and evaluate the impact of different surgical strategies on SO patient outcomes.

## CONCLUSION

In summary, when evaluating an adnexal tumor classified as O-RADS score 3-5 that exhibits features, such as a large cystic echo, regular boundaries, and signs of calcification, the possibility of SO should be considered. Moving forward, we plan to expand our sample size and refine the classification criteria, aiming to provide more robust ultrasound evidence to assist clinicians in accurately diagnosing SO.

## Figures and Tables

**Table 1:** Clinical characteristics of struma ovarii.

**Clinical Characteristics**	**Tumor Type**		**Total**
	**Cystic-solid** 13	**Cystic** 3	16
Age	-	-	-
<30	1	2	3
30~39	1	0	1
40~49	3	1	4
≥50	8	0	8
Tumor location	-	-	-
Right	6	1	7
Left	7	2	9
CA125	-	-	-
Normal	10	3	13
Elevated	3	0	3
Pelvic effusion	-	-	-
Present	4	3	7
Absent	9	0	9
Menopausal status	-	-	-
Postmenopausal	6	0	6
Premenopausal	7	3	10

**Table 2:** Ultrasonographic features of struma ovarii.

Ultrasonographic Features	Tumor Type		Total
	Cystic-solid 13	Cystic 3	16
Size	-	-	-
Maximum diameter (cm)	10	30	-
Mean (cm)	7	14	-
>10 cm	0	2	2
Smooth margins	-	-	-
Yes	13	3	16
No	0	0	0
Blood flow (Adler grade)	7	2	9
0	0	3	3
1	6	0	6
2	1	0	1
3	6	0	6
Calcification	-	-	-
Present	13	1	14
Absent	0	2	2
Septations	-	-	-
Present	10	1	11
Absent	3	2	5
O-RADS score	-	-	-
3	6	0	6
4	5	3	8
5	2	0	2

## Data Availability

All the data and supporting information are provided within the article.

## LIST OF ABBREVIATIONS

SO: Struma ovarii
TPO: Thyroid peroxidase
TVUS: Transvaginal ultrasound
O-RADS: Ovarian-Adnexal Reporting and Data System
ACR: American College of Radiology
CT: Computed tomography
MRI: Magnetic resonance imaging
MSI: Microsatellite instability

## STANDARDS OF REPORTING

STROBE and REMARK guidelines were followed.
